# Obesogenic Diets Composition Differentially Alters the *Clostridium*/*Bacteroides* Ratio and Drives Colonic Inflammation

**DOI:** 10.3390/nu18152471

**Published:** 2026-07-30

**Authors:** Mayra Montecillo-Aguado, Esmeralda Rodríguez-Miranda, Guillermina Baay-Gúzman, Juana Rosalba Garcia-Ramirez, Daniel Hernández-Cueto, Sergio López-Briones, Marco Antonio Hernández-Luna

**Affiliations:** 1 Laboratorio de Biomedicina Traslacional, Departamento de Medicina y Nutrición, Universidad de Guanajuato, León 37670, Guanajuato, Mexico; mayramontecillo@ugto.mx (M.M.-A.); erodriguez@ugto.mx (E.R.-M.); lobrisug@ugto.mx (S.L.-B.); 2 Unidad de Investigación en Enfermedades Oncológicas, Hospital Infantil de México, Federico Gómez, Ciudad de México 06720, Mexico; guillebaay@hotmail.com (G.B.-G.); danteligueri@gmail.com (D.H.-C.); 3 Departamento de Medicina y Nutrición, Universidad de Guanajuato, León 37670, Guanajuato, Mexico; jr.garcia@ugto.mx

**Keywords:** obesogenic diets, colon, inflammation, mucins, intestinal microbiota, *Clostridium*, *Bacteroides*

## Abstract

Background: Diets high in fat and carbohydrates, like fructose, trigger colon inflammation, increase intestinal permeability, and drive dysbiosis. However, the effects of obesogenic diets on gut microbiota, including *Clostridium* and *Bacteroides*, remain unknown. Understanding how these diets damage the colon is critical. Methods: Using a controlled preclinical obesity model, we compared the effects over time of High-Fat Diet (HFD), High-Fructose Diet (HFrD), and their combination (HFHFrD). Diet-induced dysbiosis was assessed at 4 and 8 weeks via qPCR using primers specific to bacterial phyla and species. In addition, intestinal inflammation, atrophy, and mucin production were evaluated by digital pathology after 8 weeks of diet exposure. Results: both HFrD and HFHFrD mice exhibited marked intestinal inflammation, atrophy, and damage, alongside altered production of neutral and mixed mucins. HFD-fed mice displayed a 15-fold surge in *Clostridium*/*Bacteroides* ratio at 4 weeks. At 8 weeks, HFrD-fed mice showed a striking 10-fold rise in microbial relative abundance compared to the other diets. Both HFrD and HFHFrD triggered an early increase in *Bacteroides* species, but significance emerged only at 8 weeks. Conclusions: Although all obesogenic diets induced inflammation, atrophy, epithelial damage, and altered mucin patterns, HFrD and HFHFrD caused pronounced disruptions to barrier function and dysbiosis. Critically, HFD consistently raised the Firmicutes/Bacteroidetes ratio at 4 and 8 weeks, while the *Clostridium*/*Bacteroides* ratio spiked only at 4 weeks. Obesogenic diets fundamentally shifted microbial load and diversity. Therefore, bacterial ratios, such as *Clostridium*/*Bacteroides*, may signal dysbiosis and tissue damage from obesogenic diets, but further research is required for confirmation.

## 1. Introduction

Obesity is a chronic disease characterized by excess body fat. It has been associated with an increased risk of cardiovascular and metabolic diseases, infertility, osteoarthritis, cancer, and other conditions [1]. One of the main causes of obesity is the excessive consumption of diets that promote weight gain, coupled with decreased physical activity. Obesogenic diets typically contain high levels of saturated fats, simple carbohydrates, and refined sugars [2]. Obesogenic diets, such as the high-fat diet (HFD) and the high-fructose diet (HFrD), have been reported to induce alterations in the gut, such as intestinal mucosal damage, by increasing permeability and triggering local inflammation [3], as well as promoting intestinal dysbiosis [4,5].

It is well known that dysbiosis induced by obesogenic diets reduces the diversity of certain bacterial species and the total microbial load [4,6]. In humans and mouse experimental models, changes in major bacterial phyla—such as increased Firmicutes and decreased Bacteroidetes—are linked to obesity [7,8]. Thus, Firmicutes/Bacteroidetes ratio has been suggested as a potential biomarker of dysbiosis.

However, the Firmicutes/Bacteroidetes ratio depends on host genetics, age, and diet; therefore, it remains a subject of debate [9,10]. Furthermore, to date, there is no conclusive information on which bacteria are affected by the consumption of high-fat or high-fructose diets, nor is there certain knowledge about the effect at higher taxonomic levels on significant changes at the bacterial genus and species levels. Thus, interesting findings have been reported regarding HFD and HFrDs when administered separately. HFD can alter intestinal bacterial communities by increasing both *Clostridium* species and the production of secondary bile acids (SBAs) [11]. Conversely, a decrease in *Clostridium* species abundance has also been observed in mice with HFD-induced obesity [12]. Regarding HFrD, it has also been observed to modify the gut microbiota, causing inflammation, and increasing intestinal permeability [13,14]. Furthermore, previous studies demonstrate that the intake of HFrD can alter barrier function, reducing mucus thickness and increasing microbiota translocation, which in turn raises blood endotoxin levels [15,16,17,18].

Therefore, maintaining a balanced gut microbiota is crucial for a healthy life, as changes in its composition can cause health problems, including inflammatory bowel disease (IBD) [19], and are a risk factor associated with colon cancer [20,21]. In this regard, an increase in the abundance of *Clostridium* species has been found in ulcerative colitis [22] and inflammatory bowel disease (IBD) [23], promoting severe inflammation and intestinal damage [24]. Conversely, supplementation with some *Bacteroides* species has been reported to reduce inflammation in experimental models of colitis [25,26]. It has been proposed that the protective mechanism of *Bacteroides* is its ability to produce short-chain fatty acids (SCFAs) [27]. SCFAs are very important for maintaining the gastrointestinal barrier because they provide energy to the epithelial cells of the colon and reduce inflammatory processes [28,29]. However, the impact of obesogenic diets on the gut microbiota, especially on the *Clostridium/Bacteroides* ratio, which produces SBAs and SCFAs, is not yet fully understood. Nevertheless, an increased risk of systemic lupus erythematosus has been associated with elevated *Bacteroides* levels and reduced *Clostridium* levels [30]. Thus, alterations in the *Clostridium*/*Bacteroides* ratio can regulate colonic epithelial functions by modifying microbial metabolite profiles. For example, increased *Clostridium* abundance has been associated with greater production of SBAs, which can disrupt the colonic epithelial barrier and promote inflammation [31], while increased *Bacteroides* levels can promote the generation of SCFAs, such as butyrate, which maintain mucosal integrity and promote anti-inflammatory mechanisms [32].

Therefore, it is interesting to study the alteration in the *Clostridium*/*Bacteroides* ratio induced by obesogenic diets. While it is well known that obesogenic diets alter and damage the intestinal barrier, it is still unknown whether this damage depends on the relative proportions of *Clostridium* and *Bacteroides*. In this study, we have performed a time series comparison of the HFD, HFrD, and a combination of both (HFHFrD) and determined how they affect the *Clostridium*/*Bacteroides* ratio, as well as the impact on inflammation, tissue integrity, and intestinal function.

## 2. Materials and Methods

### 2.1. Mice

Eight-week-old male C57BL/6 mice were acquired and housed in the Health Sciences Division animal facility at the University of Guanajuato. All mice were maintained in a pathogen-free environment under controlled conditions: 20 ± 2 °C, 50 ± 5% humidity, and a 12 h light–dark cycle. They had unrestricted access to food and water. All experiments were conducted in accordance with the official Mexican guidelines for the use and care of experimental animals. The protocol was approved by the Ethics Committee of the University of Guanajuato (CEPIUG-P53-2023).

### 2.2. Dietary Intervention, Body Weight Gain, and Colon Collection

Four groups of five mice were kept in separate cages and fed *ad libitum* for eight weeks with one of the following diets: (a) Control Diet (CD, Lab Rodent Diet 5001) (St. Louis, MO, USA), (b) High-Fat Diet (HFD, Research Diet, D12492 containing 60% fat) (New Brunswick, NJ, USA), (c) High-Fructose Diet (HFrD, a mixture of Lab Rodent Diet 5001 and 30% fructose in water), and (d) High-Fat and High-Fructose Diet (HFHFrD, a combination of Research Diet, D12492 with 60% fat and 30% fructose in water). The caloric content and composition of each diet are presented in Table S1. The average weekly food consumption for each group is shown in Supplementary Figure S1.

To assess weight gain, each mouse was weighed and recorded weekly throughout the experiment. For microbiota analysis, fecal samples were collected at the beginning of the study and again at four and eight weeks after dietary exposure. Eight weeks post-dietary exposure, blood samples were obtained from the retroocular plexus using a capillary microtube and plasma glucose levels were immediately analyzed with an Accutrend GCT system (Roche Diagnostics GmbH, Mannheim, Germany), following the manufacturer’s instructions. Subsequently, all mice were euthanized via cervical dislocation, and the colon was extracted and placed in cold phosphate-buffered saline (PBS). After several washes with cold PBS to eliminate intestinal contents, the length of the colon was measured using a conventional ruler, and the total weight of the tissue was recorded with an analytical balance. Finally, the entire colon was fixed by immersion in 4% paraformaldehyde and embedded in paraffin for further histological analysis.

### 2.3. Analysis of Colon Inflammation and Tissue Damage by Digital Pathology

For histological analysis, the colons were fixed in formaldehyde and dehydrated by treatment with xylene and ethanol (J.T. Baker, Radnor, PA, USA). The colon tissues were then embedded in paraffin, and four µm sections were cut from the embedded tissues using a rotary microtome (Leica BioSystems™, Wetzlar, Germany). These tissue sections were mounted on glass slides, deparaffinized, and stained with hematoxylin and eosin (H&E). The stained sections were digitized with an Aperio ScanScope CS2 (Leica BioSystems, Nussloch, Germany). For evaluating inflammation, atrophy, and damage to the intestinal architecture of the colon, high-resolution 20× digital images (0.45 µm/pixel) were obtained. The images were visualized and analyzed using ImageScope software version 12.4 (Leica BioSystems, Nussloch, Germany). An expert pathologist randomly selected at least three different areas from each sample and conducted a histomorphological evaluation according to Table S2 (modified from Erben et al., 2014) [33].

Thus, images of colon tissue sections stained with hematoxylin and eosin (H&E) were utilized to assess the degree of intestinal inflammation, atrophy, and damage (IAD score). The total score for each colon was calculated by adding individual scores for inflammatory cell infiltration, epithelial changes, and damage to the intestinal mucosal architecture [33]. Additionally, the lengths of the mucosal and muscularis layers were measured across 20 different fields of colonic tissue. The data were expressed as mean ± standard error (SE).

### 2.4. Evaluation of Mucin Expression Patterns and Quantification of Globet Cells via Digital Pathology

Using Periodic Acid–Schiff and Alcian Blue (PAS-AB) staining, we assessed mucus production and quality, following standard procedures. The stained tissues were scanned at 20× magnification using an Aperio ScanScope CS2 whole slide scanner (Leica BioSystems, Nussloch, Germany). For analysis, the colon tissue was divided into two central regions: proximal and distal. The Aperio positive pixel counting algorithm (version 9.2, Leica Biosystems) was employed to evaluate mucus quality in the scanned images. The inputs were pre-programmed to quantify pink, purple, and blue colors.

### 2.5. Determination of Phyla and Bacterial Species via qPCR

To determine the effects of different diets on bacterial phyla and species within each group of mice, fecal samples were collected at baseline and at four and eight weeks after dietary exposure. All fecal samples were homogenized in 500 µL of TRIzol, (Invitrogen™, Waltham, MA, USA) through sonication for 5 cycles of 30 s at 4 °C in a VC50 ultrasonic processor (Sonics & Materials, Inc., Newtown, CT, USA). DNA was then purified following the manufacturer’s protocol. To ensure DNA integrity, all samples were subjected to electrophoresis on 0.8% agarose gels using TAE buffer (40 mM Tris-acetate and 1 mM EDTA, pH 8.3). Subsequently, DNA quantification was carried out using spectrophotometry with an m200 Infinite Pro microplate reader (Tecan, Männedorf, Switzerland). Finally, 100 ng of DNA was used for qPCR assays with the SYBR Green qPCR master mix (MedChemExpress, Monmouth Junction, NJ, USA) according to the manufacturer’s instructions. Table 1 and Table 2 show the specific primers used for the phylum and bacterial species, respectively. For each of the following phyla: Firmicutes, Bacteroidetes, Actinobacteria, and Proteobacteria; the forward primers were designed in the variable region, while the reverse primer was the same one used for amplifying the conserved region of the 16S ribosomal subunit, as indicated in Table 1 and Supplementary Figure S2. DNA from the 16S rRNA subunit was used as a housekeeping control. The qPCR assays were conducted using a CFX Opus 96 Real-Time PCR system (Bio-Rad, Hercules, CA, USA). The results were calculated using the ΔCt method. Thus, the total DNA expression of each phylum or bacterial species was compared to the DNA expression of the 16S rRNA subunit as the housekeeping control. Data collection and analysis were performed using CFX Maestro software version 2.0 (Bio-Rad, Hercules, CA, USA). To ensure reproducibility of the results, all experiments were conducted in triplicate.

### 2.6. Statistical Analysis

Statistical analysis was performed using GraphPad Prism version 8.0 (La Jolla, CA, USA). The Kruskal–Wallis test followed by Dunn’s post hoc test was used for comparisons involving more than two groups. Differences between the two independent groups were assessed using the Mann–Whitney U test. All data are presented as mean ± standard deviation (SD) or standard error of the mean (SEM). Levels of statistical significance are indicated as follows: * *p* ≤ 0.05, ** *p* ≤ 0.01, *** *p* ≤ 0.001.

## 3. Results

### 3.1. Effect of Obesogenic Diets on Body Weight, Plasma Glucose and Colon Length and Weight

**Figure 1:**
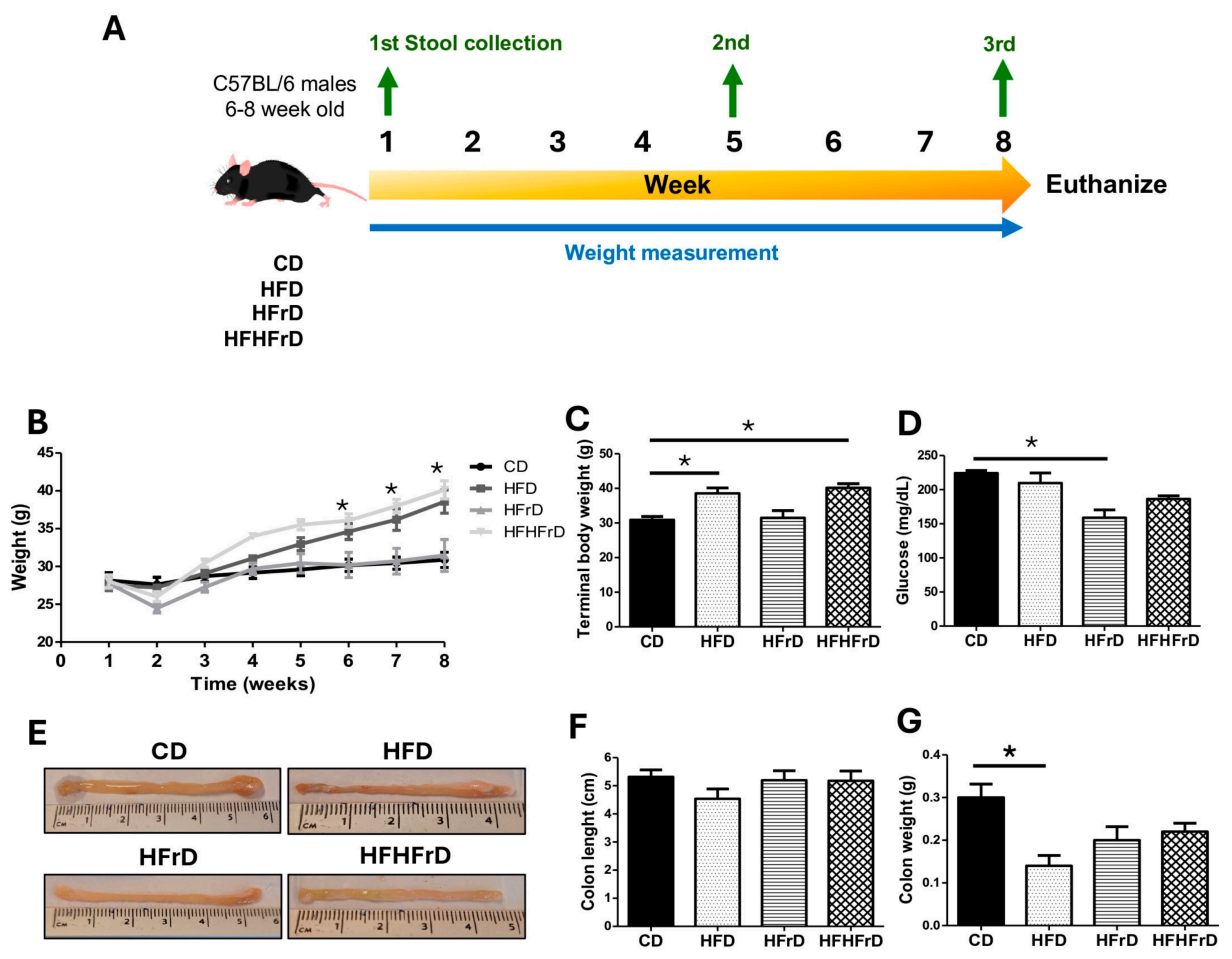
Experimental Design, Body Weight Gain, and Measurements of Plasma Glucose, Colon Length, and Weight. (**A**) Groups of five male C57BL/6 mice were fed a control diet (CD), High-Fat Diet (HFD), High-Fructose Diet (HFrD), or High-Fat/High-Fructose Diet (HFHFrD) for eight weeks. Weight gain was recorded weekly, and fecal samples were collected at baseline and at 4 and 8 weeks for microbiota analysis by quantitative polymerase chain reaction (qPCR). After the eight-week dietary exposure period, all mice were euthanized, and the colon was excised for subsequent histological analysis by digital pathology. (**B**) Body weight gain was measured weekly during eight weeks of dietary exposure. (**C**) Body weight gain and (**D**) plasma glucose levels at the end of the experiment. (**E**). Representative images of the colons of mice fed different diets are shown. In graphs (**F**,**G**), the measurements of colon length (in cm) and weight (in grams) are represented, respectively. In all graphs, data represent the mean ± SEM (*n* = 5 mice/group) and were analyzed using the Kruskal–Wallis test and Dunn post hoc comparisons. Asterisks (*) indicate statistically significant differences between groups, with * *p* < 0.05.

Four groups of five mice were fed *ad libitum* for eight weeks with one of the following diets: (a) Control Diet (CD), (b) High-Fat Diet (HFD), (c) High-Fructose Diet (HFrD), and (d) High-Fat and High-Fructose Diet (HFHFrD). The mice were weighed weekly, and fecal samples were collected at baseline and at 4 and 8 weeks, as outlined in the experimental design (Figure 1A). As expected, after eight weeks of treatment, the body weight of the HFD mice increased from 27.5 ± 1.1 g to 38.6 ± 1.7 g. Similarly, the weight of the HFHFrD mice increased from 28.0 ± 1.3 g to 40.1 ± 1.9 g. In contrast, there was no significant increase in body weight for either CD or HFrD mice (Figure 1B). Consequently, at the end of the treatment period, there was a significant difference in body weight gain between the HFD (38.6 g) and HFHFrD (40.1 g) mice compared to the CD (30.8 g) and HFrD (31.4 g) mice, respectively (Figure 1C). Remarkably, while plasma glucose levels remained within normal ranges across all four diets, a significant decrease in plasma glucose was observed in the HFrD mice compared to the CD mice. Plasma glucose levels, however, were similar in the CD, HFD, and HFHFrD mice (Figure 1D).

On the other hand, after several washes with cold PBS to remove intestinal contents, the colon length and weight were measured using standard methods. For colon length, we observed no significant differences among the four different diets, as shown in Figure 1E,F. However, it showed a significant decrease in colon weight in HFD compared with CD mice. In contrast, there were no significant differences in colon weight between HFrD, HFHFrD, and CD mice (see Figure 1G).

### 3.2. Obesogenic Diets Can Lead to Damage, Inflammation, and Atrophy of Colon Tissue

**Figure 2:**
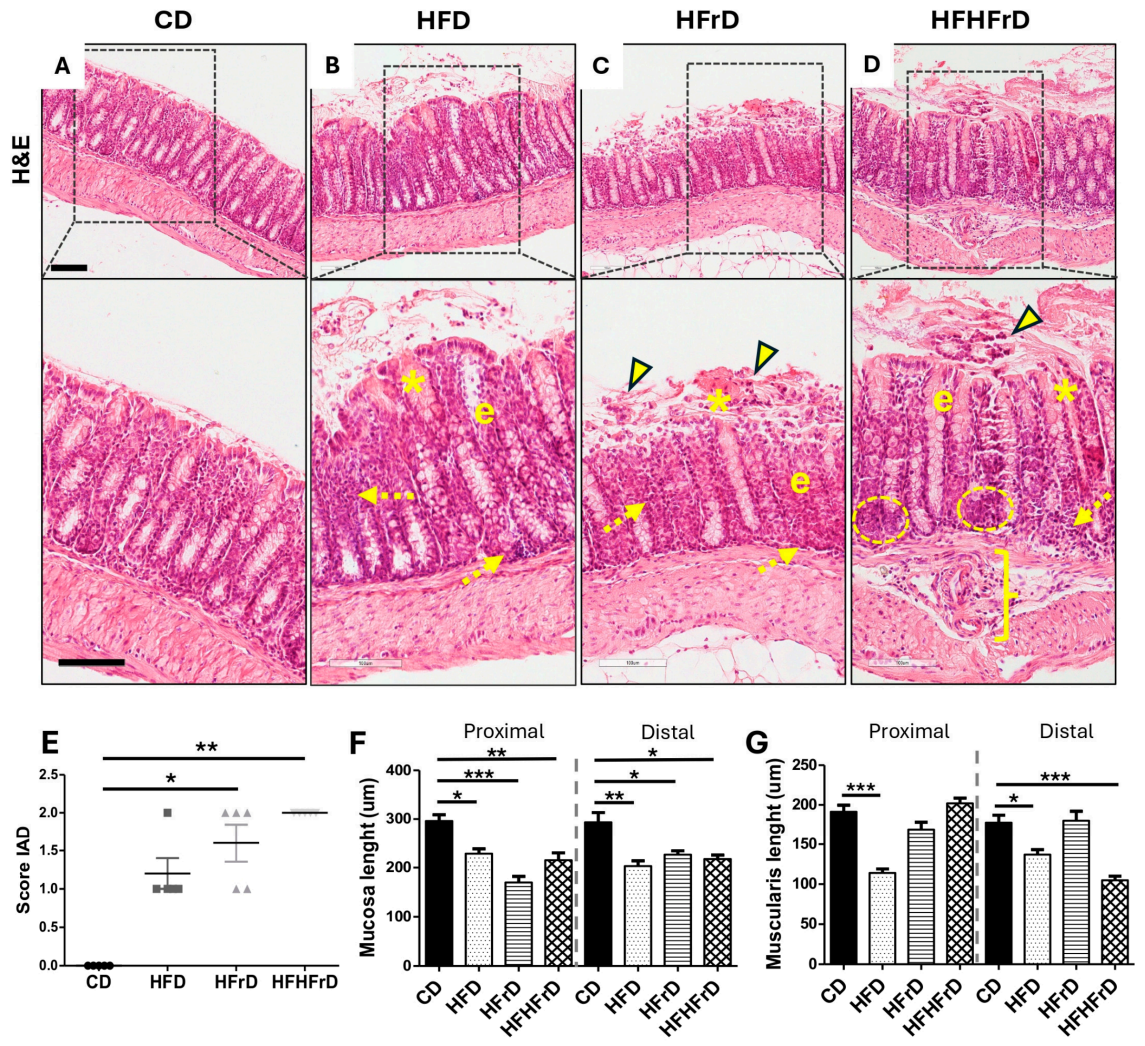
Impact of obesogenic diets on intestinal inflammation, atrophy, and colonic structural damage. Representative images of hematoxylin and eosin-stained colon tissue sections from mice subjected to different diets: CD, HFD, HFrD, and HFHFrD are shown in panels (**A**–**D**) (at **top**). A close-up of the corresponding selected section is shown at the bottom of each panel. To evaluate inflammation, atrophy, and damage to the colon’s intestinal architecture, high-resolution digital images (20× magnification, 0.45 µm per pixel) were captured and analyzed using ImageScope software. Dotted arrows and brackets were employed to highlight changes in the mucosa and submucosa, respectively. The presence of lymphoid aggregates was marked with a circle. Additionally, epithelial, and architectural changes are indicated, including mucosal edema (e), while mucosal sloughing and erosion are denoted by an arrowhead and asterisk (*), respectively. The scale bar represents a length of 100 μm. In section (**E**), the scores for intestinal inflammation, atrophy, and damage (IAD) are presented. Additionally, the measurements of mucosal length (**F**) and muscular layer length (**G**) in millimeters are included for both proximal and distal sections. In all graphs, data represent the mean ± SEM (*n* = 5 mice/group) and were analyzed using the Kruskal–Wallis test and Dunn post hoc comparisons. Asterisks (*) indicate statistically significant differences between groups, with * *p* < 0.05, ** *p* < 0.01 and *** *p* < 0.001.

To assess the effects of obesogenic diets on potential colon damage, we examined histological changes using the modified Erben Histomorphological Guide (see Table S2 in the Supplement). Images of Hematoxylin/Eosin (H&E)-stained colon tissue sections were analyzed to obtain an intestinal Inflammation, Atrophy, and Damage (IAD) score, as described in the materials and methods section. As anticipated, no inflammatory cell infiltration was observed in CD mice. Both the intestinal mucosa and epithelium appeared normal and showed no significant damage, resulting in a total score of 0 (Figure 2A,E). In contrast, the mice on an HFD exhibited mild mucosal edema and atrophy, along with a mild, scattered infiltration of inflammatory cells. This resulted in a total score of less than 2 (Figure 2B,E). Similar findings were observed in the HFrD mice; however, areas with diffuse inflammatory infiltrate, mild architectural loss, and focal detachment of the sloughed surface epithelium were also identified (arrowhead). Additionally, in some regions of the colon, focal epithelial ulcers accompanied by mild transmural edema and mucosal fold formation were noted, with a total score of 2 or less (Figure 2C,E). Moreover, the colon of HFHFrD mice showed increased focal areas of atrophy, epithelial erosion, and mucosal fold formation. Inflammatory cells were also present in the mucosa and submucosa, with lymphoid aggregates, resulting in a total score of 2 (see Figure 2D,E).

Importantly, when evaluating the thickness of the mucosal and muscularis layers in the proximal and distal colon, a significant reduction of approximately 100 μm was observed in both sections of the mucosal layer in HFD, HFrD, and HFHFrD mice (Figure 2F). Additionally, a significant decrease in the length of the muscularis layer was found in both the proximal and distal sections of the colon in HFD compared to CD mice (Figure 2G). In the HFrD mice, no significant differences in the length of the muscularis layer were observed in either the proximal or distal colon compared to CD mice. While HFHFrD mice showed no significant change in the length of the muscular layer in the proximal section, a significant reduction was noted in the distal colon when compared to CD mice (Figure 2G). These results indicate that all obesogenic diets cause varying levels of damage to the colonic epithelium, with high-fructose diets such as HFrD and HFHFrD causing greater impairment of gut barrier function.

### 3.3. HFrD and HFHFrD Mice Show Increased Production of Neutral and Mixed Mucins

In inflammatory bowel diseases, colon inflammation contributes to disrupting barrier function and altering mucin expression. Also, obesogenic diets cause inflammation, but their association with intestinal barrier damage and altered mucin expression remains poorly understood.

**Figure 3:**
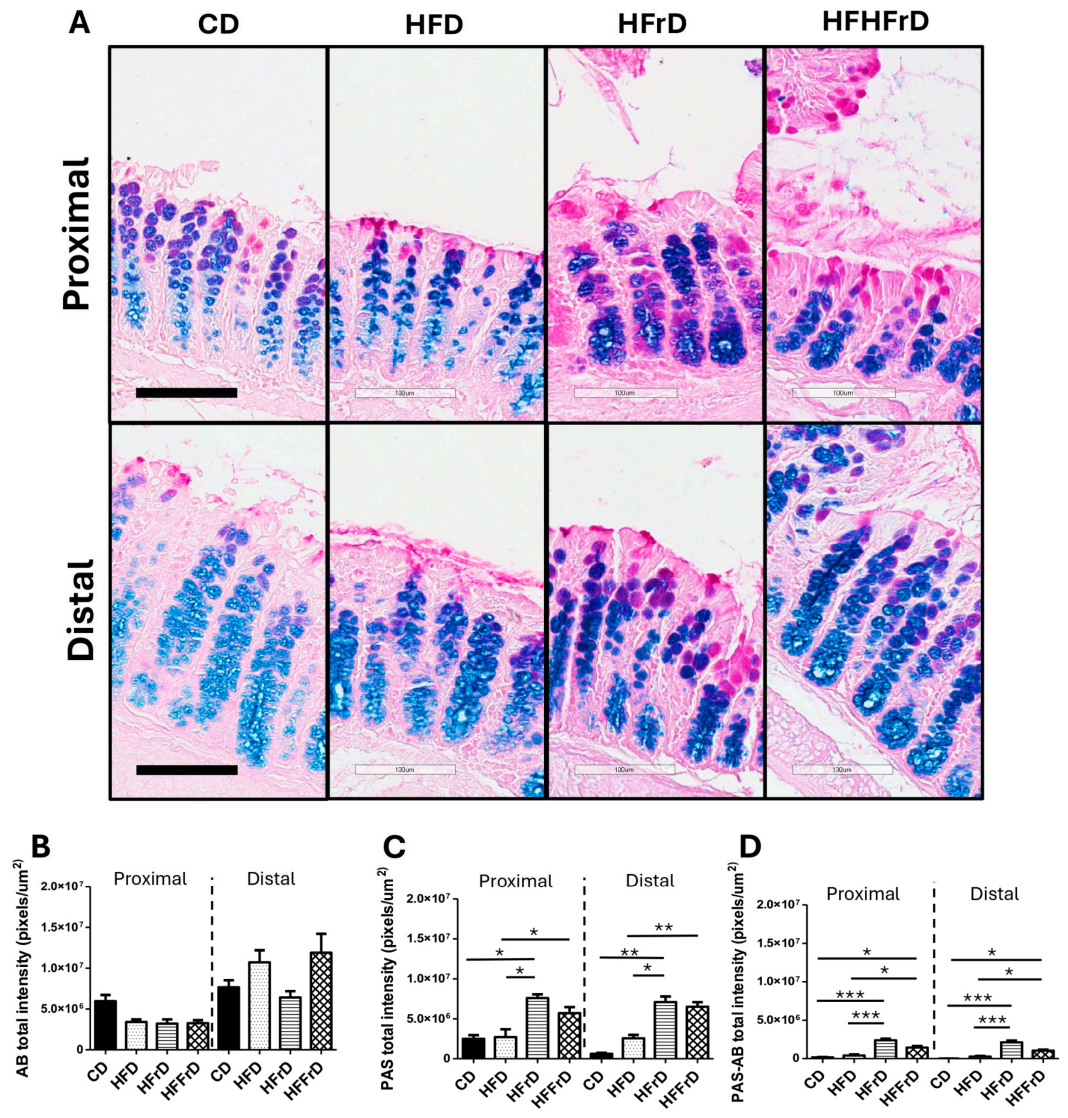
Obesogenic diets alter mucin production in the colon. PAS-AB staining was used to assess the effects of obesogenic diets on mucin expression in the colon. (**A**) The staining patterns of mucin-producing goblet cells in the colon of mice fed four different diets are shown, analyzed in both the proximal (**top**) and distal (**bottom**) regions. The scale bar represents a length of 100 μm. (**B**) The expression of Alcian blue (AB)-positive acidic mucins is depicted, and the graph quantifies the intensity of Alcian blue staining (measured in pixels/mm^2^) in both the proximal and distal colon after exposure to different diets. (**C**) Determination of the expression of neutral periodic acid–Schiff (PAS)-positive mucins in proximal and distal sections; the graph quantifies the intensity of the magenta staining (pixels/mm^2^). (**D**) Analysis of periodic acid–Schiff–Alcian blue (PAS-AB)-positive goblet cells producing mixed mucins in the proximal and distal sections of the colon. The intensity of violet staining is quantified (pixels/mm^2^). In all graphs, data represent the mean ± SEM (*n* = 5 mice/group) and were analyzed using the Kruskal–Wallis test and Dunn post hoc comparisons. Asterisks (*) indicate statistically significant differences between groups, with * *p* ≤ 0.05, ** *p* ≤ 0.01 and *** *p* ≤ 0.001.

For this reason, our next objective was to investigate how different diets could affect the expression patterns of the main mucins using digital pathology. These mucus-producing proteins play essential roles, such as providing lubrication, protecting against mechanical and chemical damage, and facilitating interactions between the host and its microbiota. To assess and characterize the effects of obesogenic diets on the mucins lining the intestinal mucosa, we used PAS-AB staining, which stains neutral mucins (PAS) and acidic mucins (AB). Figure 3A shows the staining patterns of mucin expression in the colons of mice exposed to four different diets, either in the proximal (top) or distal (bottom) regions. When examining AB-positive staining for acidic mucins, the intensity of Alcian blue staining was found to be similar in both the proximal and distal colon following exposure to different diets (see Figure 3A,B). The staining intensity of neutral mucins, indicated by PAS magenta, was similar between the CD (2.48 × 10^6^ ± 2.3 × 10^5^ pixels/mm^2^) and HFD (2.70 × 10^6^ ± 4.9 × 10^5^ pixels/mm^2^) mice in the proximal colon, and no difference was observed in the distal section either. However, there was a significant increase in PAS magenta staining intensity in HFrD (7.58 × 10^6^ ± 2.3 × 10^5^ pixels/mm^2^) and HFHFrD (5.71 × 10^6^ ± 3.8 × 10^5^ pixels/mm^2^) mice compared to CD mice. Notably, the HFD mice (2.55 × 10^6^ ± 2.4 × 10^5^ pixels/mm^2^) exhibited lower PAS magenta staining intensity than both the HFrD (7.08 × 10^6^ ± 3.3 × 10^5^ pixels/mm^2^) and HFHFrD (6.51 × 10^6^ ± 2.8 × 10^5^ pixels/mm^2^) mice in the distal colon (see Figure 3A,C). Similarly, in both HFrD (2.38 × 10^6^ ± 1.0 × 10^6^ pixels/mm^2^) and HFHFrD (1.44 × 10^6^ ± 1.1 × 10^6^ pixels/mm^2^) mice, a significant increase in mixed mucin expression was found; these cells were identified as PAS-AB positive (indicated by violet color) (Figure 3D). These findings indicate that obesogenic diets, particularly those containing fructose (HFrD and HFHFrD), increase the production of neutral and mixed mucins in both the proximal and distal regions of the colon. This leads to greater damage to the colonic epithelium and alters the interactions between the microbiota and the host.

### 3.4. Obesogenic Diets Induce Changes in Bacterial Phyla After Four and Eight Weeks of Dietary Exposure

**Figure 4:**
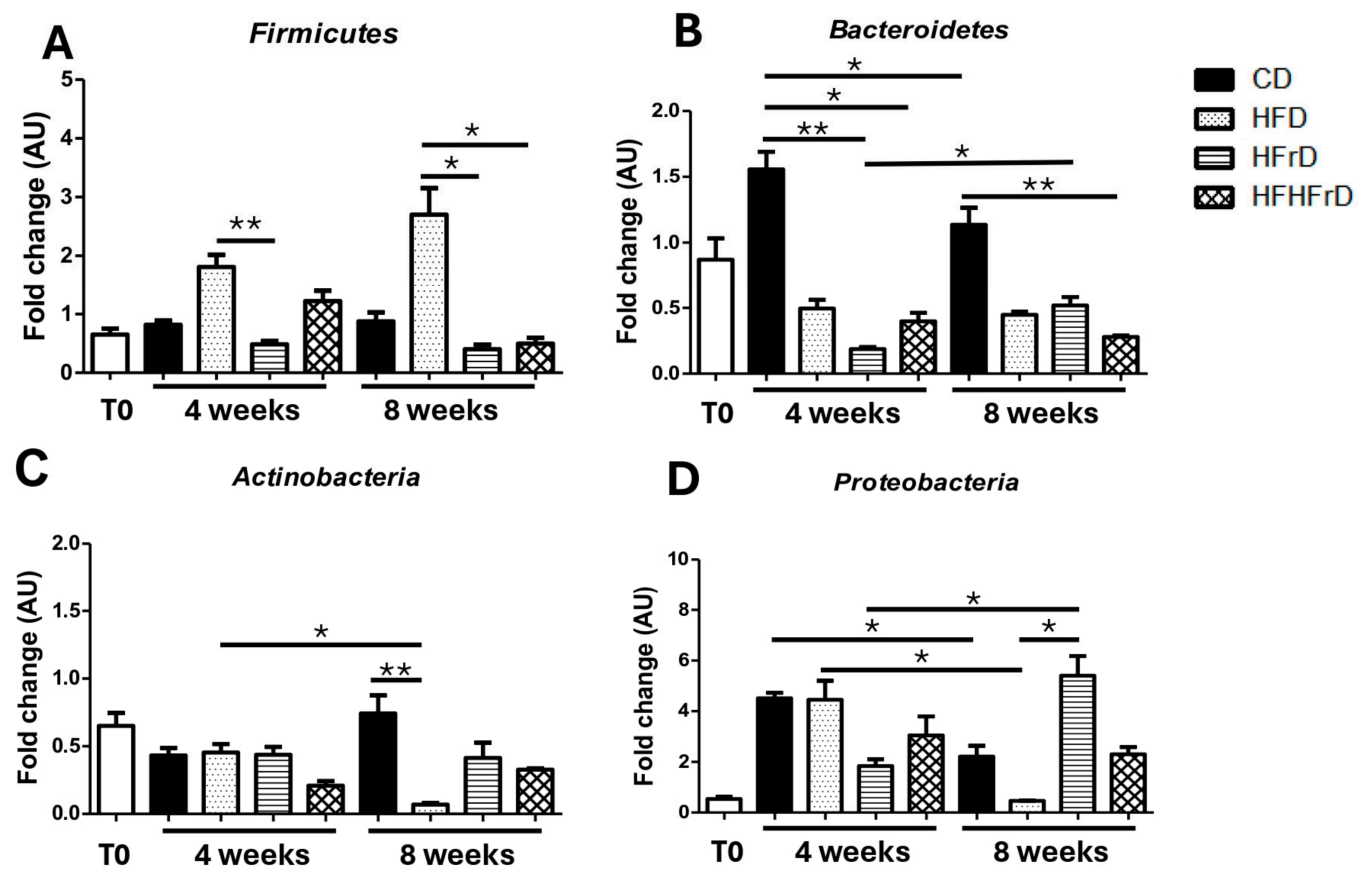
Changes in bacterial phyla after four and eight weeks of exposure to obesogenic diets. The effects of HFD, HFrD, and HFHFrD on major bacterial phyla (Firmicutes, Bacteroidetes, Actinobacteria, and Proteobacteria) were evaluated after 4 and 8 weeks of dietary exposure using qPCR, as described in the Materials and Methods. The graphs show the relative expression values (fold changes) of bacterial phyla relative to the 16S gene (housekeeping gene) using the ΔCt method, as obtained with CFX Maestro software. The Firmicutes (**A**), Bacteroidetes (**B**), Actinobacteria (**C**), and Proteobacteria (**D**) are shown, respectively. The blank bar represents the baseline (T0). Data represent mean ± SEM (*n* = 5 mice/group). Asterisks (*) indicate statistically significant differences between groups, with * *p* ≤ 0.05 and ** *p* ≤ 0.01. The Kruskal–Wallis test and Dunn post hoc comparisons were used to compare the groups by week, while the Mann–Whitney U test was used to compare the groups between four and eight weeks of dietary exposure.

So far, we have described that obesogenic diets can cause damage, inflammation, and atrophy of colonic tissue, as well as changes in the mucins that line the intestinal mucosa and interact with the host microbiota. For this reason, the main bacterial phyla in intestinal microbiota were evaluated via qPCR, in groups exposed to different diets. Thus, the effects of HFD, HFrD, and HFHFrD on the main bacterial phyla: Firmicutes, Bacteroidetes, Actinobacteria, and Proteobacteria were evaluated after four and eight weeks of dietary exposure. As shown in Figure 4A, the HFD significantly increased the levels of Firmicutes at both the four-week and eight-week marks. In contrast, no changes in Firmicutes were observed in CD or HFrD mice at either time point. In HFHFrD mice, a trend towards a decrease in Firmicutes was observed at eight weeks compared to four weeks. In HFrD mice, the Bacteroidetes phylum showed a significant decrease at four weeks compared to CD mice, although a slight increase was noted at eight weeks. Both HFD- and HFHFrD-exposed mice showed a decline at 4 and 8 weeks compared with CD mice. In CD mice, a decreasing trend in Bacteroidetes was also observed at eight weeks compared to four weeks (Figure 4B). Meanwhile, in Figure 4C, a trend toward decreased Actinobacteria in HFHFrD mice is observed at 4 weeks compared to CD, HFD, and HFrD mice. By eight weeks, both CD and HFHFrD mice show a slight increase in the Actinobacteria phylum compared to their levels at four weeks. However, at the eight-week mark, HFD mice exhibit a significant decrease in the Actinobacteria phylum compared to CD mice. In addition, Figure 4D shows that after four weeks, there were no significant differences in the Proteobacteria phylum between the CD and HFD mice. Likewise, a downward trend was observed in HFrD and HFHFrD mice when compared to CD mice. Notably, this bacterial phylum increased at 4 weeks post-exposure across all 4 diets compared to baseline. After eight weeks, a significant increase in Proteobacteria was detected in HFrD mice compared to the CD, HFD, and HFHFrD mice. Remarkably, the HFD mice exhibited a significant decrease in Proteobacteria at eight weeks compared to four weeks, while a downward trend was also detected in both CD and HFHFrD mice.

### 3.5. HFD Increases Firmicutes Phyla at Four Weeks of Dietary Treatment

**Figure 5:**
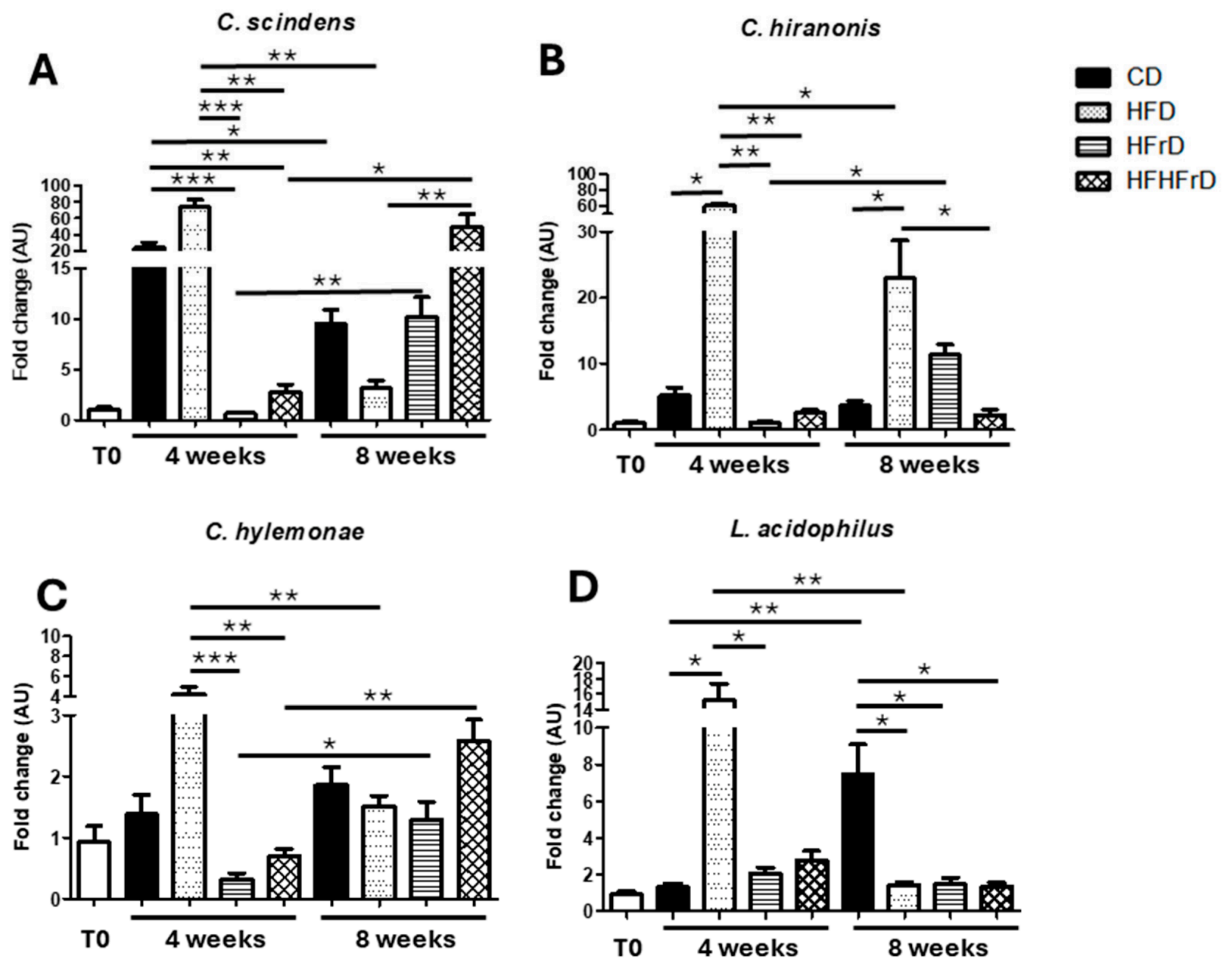
Effect of obesogenic diets on Firmicutes species at four and eight weeks of dietary treatment. The effects of HFD, HFrD, and HFHFrD on major bacterial species within the Firmicutes phylum were evaluated after 4 and 8 weeks of dietary exposure using qPCR. The graphs show the relative expression values (fold change) of the following bacterial species: *C. scindens* (**A**), *C. hiranonis* (**B**), *C. hylemonae* (**C**), and *L. acidophilus* (**D**) in relation to the 16S gene (housekeeping gene) using the ΔCt method, as obtained through CFX Maestro software. The blank bar represents the baseline (T0). Data represent mean ± SEM (*n* = 5 mice/group). Asterisks (*) indicate statistically significant differences between groups, with * *p* ≤ 0.05, ** *p* ≤ 0.01 and *** *p* ≤ 0.001. The Kruskal–Wallis test and Dunn post hoc comparisons were used to compare the groups by week, while the Mann–Whitney U test was used to compare the groups between four and eight weeks of dietary exposure.

Because significant differences in the proportions of certain bacterial phyla were observed after four and eight weeks of exposure to obesogenic diets, we evaluated several bacterial species, including Firmicutes (*C. scindens*, *C. hiranonis*, *C. hylemonae*, and *L. acidophilus*), Bacteroidetes (*B. fragilis* and *B. tethaiotaomicron*), Actinobacteria (*B. bifidum*), and Proteobacteria (*E. coli*). Thus, Figure 5A shows that at four weeks, there was a significant increase in the proportion of *C. scindens* in both CD and HFD mice. In contrast, the proportions in the HFrD and HFHFrD mice remained similar to the baseline levels. Interestingly, at eight weeks, a significant increase in *C. scindens* was detected in HFHFrD compared to CD mice. When comparing the proportions of *C. scindens* at eight weeks to those at four weeks, there was a downward trend in both the CD and HFD groups, while a significant increase was found in HFHFrD mice. In addition, at four weeks a significant increase in *C. hiranonis* was also observed in HFD mice compared to CD mice, although a slight downward trend in *C. hiranonis* was detected at eight weeks. During both four and eight weeks, there were no significant differences in the proportions of *C. hiranonis* among CD, HFrD and HFHFrD mice (Figure 5B).

In contrast, at four weeks, the proportion of *C. hylemonae* significantly increased in HFD mice, while no differences were observed among CD, HFD, and HFrD mice. By eight weeks, no differences were identified among the four groups. Additionally, when comparing the proportions between four and eight weeks, no significant differences were found, although an upward trend was detected in HFHFrD mice (Figure 5C). Regarding *L. acidophilus*, intriguing findings were observed. At week four, there was a significant increase in the proportion of *L. acidophilus* in HFD mice, but this proportion decreased significantly by week eight, while at week eight, a slight increase in the proportion of *L. acidophilus* was noted in CD mice compared to week four. While in HFrD and HFHFrD mice, no differences in the proportion of *L. acidophilus* were observed at either week four or week eight (see Figure 5D).

### 3.6. The HFrD and HFHFrD Lead to an Increase in the Bacteroidetes Phyla at Four Weeks of Dietary Treatment

**Figure 6:**
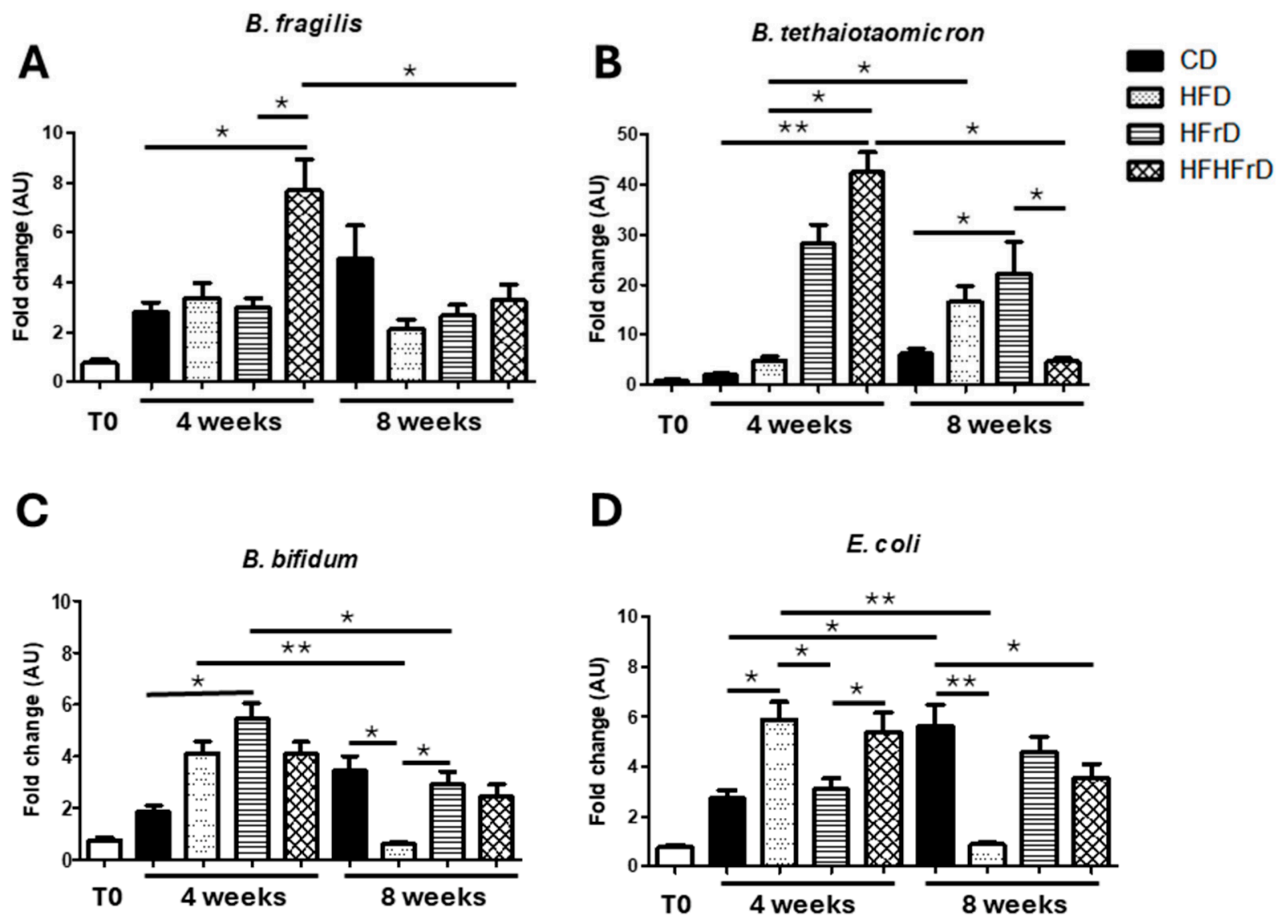
Effect of obesogenic diets on species of Bacteroidetes, Actinobacteria, and Proteobacteria after 4 and 8 weeks of dietary treatment. The main bacterial species within the phyla Bacteroidetes, Actinobacteria, and Proteobacteria were assessed after four and eight weeks of dietary exposure via qPCR. The graphs show the relative expression values (fold change) of the following bacterial species: *B. fragilis* (**A**), *B. thetaiotaomicron* (**B**), *B. bifidum* (**C**), and *E. coli* (**D**) in relation to the 16S gene (housekeeping gene) using the ΔCt method, as obtained through CFX Maestro software. The blank bar represents the baseline (T0). Data represent mean ± SEM (*n* = 5 mice/group). Asterisks (*) indicate statistically significant differences between groups, with * *p* ≤ 0.05 and ** *p* ≤ 0.01. The Kruskal–Wallis test and Dunn post hoc comparisons were used to compare the groups by week, while the Mann–Whitney U test was used to compare the groups between four and eight weeks of dietary exposure.

Additionally, the proportions of two *Bacteroides* species were analyzed. Figure 6A shows a significant increase in *B. fragilis* in HFHFrD mice at four weeks, which then decreased by eight weeks. Although no significant differences were observed in the proportion of *B. fragilis* among CD, HFD, or HFrD mice at either the four-week or eight-week mark.

Similarly, *B. tethaiotaomicron* showed a significant increase in both HFrD and HFHFrD mice at 4 weeks, whereas the proportions in CD and HFD mice remained at baseline levels. By eight weeks, the proportion of *B. tethaiotaomicron* in CD, HFD, and HFrD mice remained comparable to that at four weeks. However, HFHFrD mice exhibited a significant decrease in *B. tethaiotaomicron* at this time point (Figure 6B).

When assessing the proportions of Actinobacteria (Figure 6C) and Proteobacteria *species* (Figure 6D), a significant increase in *B. bifidum* (Actinobacteria) was observed at four weeks in HFrD mice, while no differences from baseline were observed in CD, HFD, and HFHFrD. At eight weeks, the proportions of *B. bifidum* in CD, HFrD, and HFHFrD mice remained similar to those at four weeks, but a decrease was observed in the HFD group, dropping to levels comparable to baseline (Figure 6C). Regarding *E. coli* (Proteobacteria), a significant increase was observed in both HFD and HFHFrD mice at 4 weeks, whereas *E. coli* levels remained similar to baseline in both CD and HFrD mice. At eight weeks, the proportions of *E. coli* were similar to those observed at four weeks in CD, HFrD, and HFHFrD mice, but a decrease was observed in HFD mice to levels like baseline (Figure 6D).

**Figure 7:**
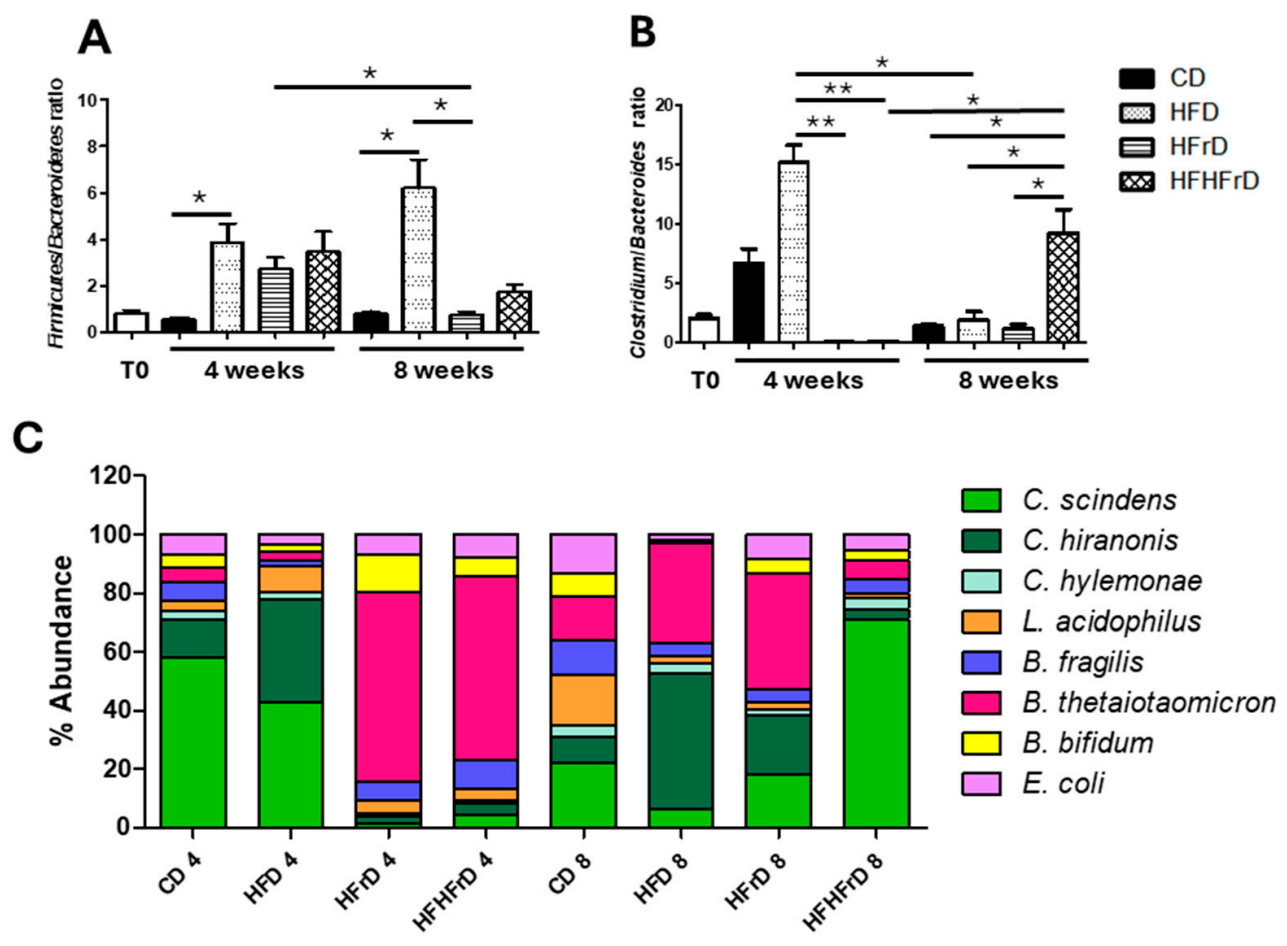
Effects of obesogenic diets on the Firmicutes/Bacteroidetes and *Clostridium*/*Bacteroides* ratios and abundance of species after four and eight weeks of dietary treatment. The ratios of Firmicutes/Bacteroidetes (**A**) and *Clostridium*/*Bacteroides* (**B**) are shown, respectively. The blank bar represents the baseline (T0). In (**C**), the distribution pattern (% of abundance) of bacterial species from each phylum is shown after four and eight weeks of dietary exposure. Each bacterial species is represented in a different color. Data represent mean ± SEM (*n* = 5 mice/group). Asterisks (*) indicate statistically significant differences between groups: * *p* ≤ 0.05, and ** *p* ≤ 0.01. The Kruskal–Wallis test and Dunn post hoc comparisons were used to compare the groups by week, while the Mann–Whitney U test was used to compare the groups between four and eight weeks of dietary exposure.

Finally, the Firmicutes/Bacteroidetes and *Clostridium*/*Bacteroides* ratios were analyzed to determine whether they exhibited the same behavior. At four weeks, HFD mice showed an increase in Firmicutes/Bacteroidetes ratio compared to CD mice, while both HFrD and HFHFrD mice showed an upward trend as well. At eight weeks, HFD mice showed a greater increase in the Firmicutes/Bacteroidetes ratio compared to their four-week measurements. In contrast, both HFrD and HFHFrD mice showed a downward trend at eight weeks compared to four weeks (Figure 7A). Interestingly, when analyzing the ratio between *Clostridium* species/*Bacteroides* species, we found that at four weeks, the HFD also increased the ratio, but the HFrD and HFHFrD decreased it. While the CD also showed a slight increase. However, at eight weeks, HFD decreases the ratio, and only HFHFrD shows an increase compared to CD, HFrD, and the same HFD (Figure 7B). Consequently, our results show that the *Clostridium*/*Bacteroides* ratio increases in HFHFrD mice and decreases in HFD mice, depending on the dietary exposure time.

This alteration in the *Clostridium*/*Bacteroides* ratio can be observed by the change in the abundance of these species caused by exposure to different diets (Figure 7C). A significant abundance of *C scindens* and *C. hiranonis* was observed in CD and HFD mice at four weeks, whereas HFrD and HFHFrD increased the abundance of *B. tethaiotaomicron*. However, at eight weeks, the CD mice did not show a particular increase in the abundance of the species analyzed, while in the HFD, *Clostridium* species predominated. Still, an increase in *B. tethaiotaomicron* was observed. Meanwhile, the HFrD mice showed an increase in *Clostridium* species and a decrease in *B. tethaiotaomicron*. As for the HFHFrD, it showed an increase in *Clostridium* species, particularly *C. scindens*, and a decrease in *B. tethaiotaomicron*.

## 4. Discussion

Obesogenic diets have been associated with gastrointestinal tract disorders such as irritable bowel syndrome and an increased risk of colon cancer [34], but the mechanism underlying this effect remains unclear [35,36]. Currently, preclinical models in the C57BL/6 mouse strain are widely used to study the effects of obesogenic diets on obesity development, especially the High-Fat Diet-induced obesity model, in which body weight gradually increases after several weeks of dietary exposure [37]. Another experimental model involves exposing mice to a High-Fructose Diet; however, it has been reported not to cause obesity [38,39]. Although it is still unclear why fructose consumption does not cause obesity in mice.

Therefore, to determine the effect of obesogenic diets on intestinal inflammation, atrophy, damage, and dysbiosis, this study compared HFD, HFrD, and a combination of both HFHFrD in C57BL/6 mice. Our results showed that HFD and HFHFrD mice increased body weight, whereas HFrD mice did not, consistent with previous reports [38,39]. Thus, we suggest that the weight gain in HFHFrD mice is due to fat intake rather than fructose consumption [40]. Notably, a significant decrease in plasma glucose level was found in HFrD mice. Similarly, it has been reported that in HFD mice, serum glucose and cholesterol levels were like those in CD mice [41]. It is important to mention that in our experimental model, the C57BL6/j mice were fed different diets for 8 weeks, and insulin resistance assays were not performed. Insulin resistance has been described as developing and becoming established after 12 weeks of exposure to a high-fat diet (HFD) [42], and when combined with fructose, insulin resistance may be slightly accelerated [43], although there is no conclusive data on this. On the other hand, when streptozotocin is used to induce diabetes, insulin resistance appears rapidly, but this is due to the destruction of pancreatic beta cells [44]. However, this experimental model is completely different from the one used here.

Moreover, a decrease in colon weight was observed only in HFD mice, suggesting an indirect effect of chronic inflammation and colon tissue damage. However, no apparent changes in colon length were observed with any dietary treatment, contradicting a previous report indicating a decrease in colon length in HFD or HFrD mice [45,46]. However, the differences may be due to differences in the duration of dietary exposure.

Furthermore, it was described here that colonic tissue damage, inflammation, and atrophy were induced in mice fed obesogenic diets. However, the severity of intestinal inflammation, atrophy, and damage (IAD score) varied significantly based on the type of diet. Mice on HFD exhibited mild tissue damage and inflammation. In contrast, those on HFrD and HFHFrD displayed more pronounced damage and inflammation. Meanwhile, mice on an HFD and combined HFHFrD had the highest tissue damage scores, characterized by significantly increased mucosal edema and severe inflammation. In this sense, a very important limitation of our study is that, based on the results obtained, we cannot clearly establish a specific mechanism for the effect of different diets on changes in the intestine, such as intestinal impermeability, inflammation, detachment, etc.

Additionally, both HFD and HFrD consumption have been shown to disrupt the colonic intestinal barrier through distinct mechanisms, although these mechanisms remain incompletely understood [18,47,48]. It has been suggested that the damage associated with an HFD may be linked to the increased production of reactive oxygen species (ROS) triggered by fatty acids and deoxycholic acid. This process promotes the synthesis and activation of myosin light chain kinase (MLCK), ultimately leading to reduced tight junctions (TJs) and zonula occludens-1 (ZO-1) [49,50]. Similarly, the effects of HFrD on tissue damage are not well understood. However, it has been suggested that this diet reduces the production of Short-Chain Fatty Acids (SCFAs), such as butyrate. These SCFAs play a crucial role in the metabolism and assembly of tight junction (TJ) proteins in the colonic epithelium [51,52]. Additionally, HFrD may reduce the synthesis and expression of Occludin and ZO-1 proteins, leading to increased inflammation [15,53]. In addition, it has been reported that HFD decreases the expression of Jam2, Claudin 1, and Claudin 3 [54]. Likewise, it has been reported that this diet increases IL-6 expression [55], an interleukin associated with increased Claudin 2 expression [56]. This contributes to claudin switching, in which the expression of claudins that form the epithelial barrier, such as Claudins 1 and 3, decreases, while the expression of pore-forming claudins, such as Claudin 2, increases [57]. These changes disrupt tight junction integrity by reducing the abundance of sealing proteins and increasing channels that allow paracellular passage of solutes and water, thereby directly increasing intestinal permeability. Furthermore, studies in various animal models have reported that fructose exposure decreases the expression of ZO-1, Claudin-1, and Occludin [58,59]. Because these proteins are critical components of the tight junction complex, their reduction weakens the junctional seal between epithelial cells, further facilitating the passage of luminal contents into the mucosa. For its part, the combination of fat and fructose in the diet reduces the expression of Occludin and ZO-1 and promotes the release of endotoxigenic LPS, which activates the TLR4-NF-κB pathway and increases the proinflammatory cytokines IL-1β, interferon-γ, and TNF-α, thereby increasing intestinal permeability [60]. Furthermore, the decrease in *Bifidobacterium spp*. and *Lactobacillus spp*. after HFD exposure has been associated with damage to, and increased permeability of, the intestinal barrier [61,62]. These microbial species have been linked to the expression of Occludin, ZO-1, MLCK, and Claudin 3 [63,64,65]. As our results show, after 8 weeks of exposure to obesogenic diets, we observed a decrease in *B. bifidum* and *L. acidophilus* (Figure 5D and Figure 6C); however, more *Bifidobacteria* and *Lactobacillus* species must be evaluated to determine their role in controlling the damage and permeability of the intestinal barrier caused by obesogenic diets.

Thus, our findings align with previous data, as we observed increased inflammation and tissue damage in the colon of mice exposed to either HFrD or HFHFrD compared to those fed HFD. However, future experiments are needed to evaluate intestinal permeability and changes in tight junction proteins to establish a specific mechanism of tissue damage induced by exposure to different diets. Likewise, exposure to an HFrD has been shown to significantly increase proinflammatory cytokines, such as IL-1β, IL-6, IL-8, TNF-α, and Macrophage Inflammatory Protein 2 (MIP-2) [3,53,66]. This increase in cytokines is associated with a reduction in colon length because of inflammation and colonic tissue damage [46]. Also, HFD has been linked to inflammation in colonic epithelium, driven by a significant increase in cytokines and chemokines [3,67]. Additionally, prolonged consumption of saturated fats has been reported to contribute to low-grade chronic inflammation [68].

On the other hand, alterations in the quantity and quality of intestinal mucus are linked to colon inflammation, as these changes facilitate contact between bacteria and intestinal epithelial cells, thereby increasing the host’s immune response and exacerbating inflammation [36,69]. In this study, we found that mice fed HFrD or HFHFrD exhibited increased numbers of mucus-producing goblet cells and increased neutral mucin production in both the proximal and distal regions of the colon. Furthermore, prolonged consumption of an HFD has been shown to alter mucin oligosaccharide chains, thereby affecting their structure and availability [70]. In a related manner, HFrD has been shown to increase the population of *Akkermansia muciniphila* (*A. muciniphila*), a bacterium that actively degrades the mucus layer [71]. However, in our study, we found no significant differences in the production of acidic mucins between the different diets.

Numerous studies have shown that diets high in fat and/or fructose can disrupt the balance of gut microbiota [72,73,74]. Specifically, an HFD is associated with an increased Firmicutes/Bacteroidetes ratio [75], whereas an HFrD tends to decrease this ratio [76]. Consistent with previous findings, we observed that the Firmicutes/Bacteroidetes ratio began to increase in HFD mice at 4 weeks, with a more pronounced increase at 8 weeks. When the effects of HFrD and HFHFrD on the Firmicutes/Bacteroidetes ratio were evaluated, a slight increase was found at four weeks, followed by a significant decrease at eight weeks. Our results clearly suggest that fructose had a greater impact on decreasing the Firmicutes/Bacteroidetes ratio than fat.

On the other hand, reports indicate that HFD increases the abundance of Proteobacteria [77]. This finding contradicts our results, which show a decrease after eight weeks of exposure to an HFD. In contrast, exposure to fructose increased the Proteobacteria population over the same period. This finding is consistent with previous reports indicating that ingestion of both fructose and glucose increases the Proteobacteria population in mice treated for 12 weeks [59]. Notably, a significant decrease in Actinobacteria in HFD mice was observed at 8 weeks, whereas no significant changes were detected with the other diets.

Controversy persists regarding how obesogenic diets affect the Firmicutes phylum. This phylum includes the family Clostridiaceae, with species such as *C. scindens, C. hiranonis*, and *C. hylemanae*, which are linked to secondary bile salt formation [78,79]. Earlier reports indicated that high-fat diets (HFD) increased Firmicutes proportions [11]. However, Wei et al. [12] found that HFD decreased *C. scindens* and *C. hylemonae* in obese-prone mice but increased them in obesity-resistant mice, highlighting a genetic influence on obesity and intestinal dysbiosis. In our study, after feeding C57BL/6 mice HFD for 4 weeks, we observed increases in *C. scindens*, *C. hiranonis*, and *C. hylemonae*; by the eighth week, only *C. hiranonis* remained elevated. In contrast, high-fructose diets (HFrD) had no effect on *Clostridium* species at either time point, while week eight HFHFrD mice showed increased *C. scindens* and *C. hylemonae*.

Likewise, species from the Bacteroidetes phylum, like *B. thetaiotamicron* and *B. fragilis*, are associated with obesity [80] and non-alcoholic fatty liver disease [81], respectively. Higher proportions of these bacteria are also linked to anti-inflammatory effects [82,83,84]. Specifically, *B. thetaiotaomicron* increases IL-10 and decreases TNF-α levels through bacterial extracellular vesicles (BEVs) [85]. and may protect against non-alcoholic fatty liver disease in murine models [86]. Additionally, *B. thetaiotaomicron* produces SCFAs, such as propionate, which are linked to reduced inflammation in mouse colitis models [87]. Our findings show that *B. fragilis* increased in HFHFrD mice at 4 weeks but decreased significantly by 8 weeks, whereas no significant changes were observed in the other diet groups at either point. At week four, *B. thetaiotaomicron* increased in HFrD and HFHFrD mice but significantly decreased in both by week eight, while HFD mice showed a significant increase. These results align with those of Townsend et al. [88], who found that carbohydrates such as glucose and sucrose reduce *B. thetaiotaomicron* abundance by regulating the Roc system.

Additionally, bacterial species such as *L. acidophilus* [89] and *B. bifidum* [90] have been extensively utilized as probiotics, and their roles in obesity have been investigated. We found that after four weeks, the proportion of *L. acidophilus* increased only in HFD mice, but by the eighth week, it decreased significantly. While *B. bifidum* levels increased with all obesogenic diets, significant changes were observed only in the HFrD mice. By the eighth week, groups fed obesogenic diets showed a significant reduction in the proportion of *B. bifidum*. This bacterial species is associated with thermogenesis in adipose tissue [90]. The gut microbiota is essential for regulating metabolism, immunity, and intestinal barrier function. Dysbiosis of gut microbiota is frequently associated with pathologies involving immune system activation and intestinal inflammation. The effects of diet on gut microbiota have been extensively studied; however, other factors can also affect its composition. External stressors, such as environmental stress, antibiotic exposure, sleep disorders, physical activity, and psychological stress, can also significantly influence gut microbiota [91]. It is important to note that this study focused solely on diet type and exposure time. Although we conducted our experiments under controlled conditions, we cannot completely rule out the possibility that other variables may influence the variability in bacterial communities.

Finally, we have determined that the changes in the phyla and the Firmicutes/Bacteroidetes ratio caused by the obesogenic diet are comparable to those previously reported [73,75,76]. However, the most important finding was that in HFrD and HFHFrD mice, a significant increase in the intestinal inflammation, atrophy, and damage (IAD) score was observed. In addition, colon damage and inflammation were accompanied by a significant increase in neutral mucins and goblet cells, which produce mixtures of acidic and neutral mucins. Importantly, a notable increase in the *Clostridium*/*Bacteroides* ratio was also observed in the HFHFrD mice after eight weeks of dietary treatment. In addition, a significant decrease in *B. thetaiotaomicron*, a bacterial species known for its anti-inflammatory effects, was observed in HFHFrD mice. Thus, we suggest that prolonged exposure to an HFHFrD can disrupt the gut microbiota balance and trigger an inappropriate inflammatory response. Like other studies [3,91], we propose that the complete digestion of dietary carbohydrates yields significant amounts of pro-inflammatory metabolites, including indoxyl sulfate, arachidonic acid, and stearic acid. These metabolites can disrupt tight junction proteins, leading to damage and increased intestinal permeability. As a result, the intestinal mucosal immune system remains continuously activated, triggering an inappropriate immune response that ultimately leads to intestinal inflammation.

Our results are interesting; however, we believe it is essential to evaluate a larger number of bacterial species, including *A. muciniphila*, which plays a significant role in intestinal mucus degradation [92]. Additionally, we employed a semiquantitative qPCR technique to assess bacterial phyla, whereas modern technologies like next-generation sequencing (NGS) have enabled a comprehensive evaluation of the changes in gut microbiota phyla and genera induced by obesogenic diets [11,75,93]. Results from semi-quantitative techniques, such as RT-qPCR and qPCR, align with the changes reported using NGS [94], affirming our results as valid and reliable. In future experiments, it will be crucial to investigate the molecular mechanisms by which an HFHFrD contributes to increased colon damage, inflammation, and dysbiosis. Moreover, it is important to note that, despite the limited sample size, we found statistically significant differences in the Firmicutes/Bacteroidetes and *Clostridium*/*Bacteroides* ratios after 4 and 8 weeks of dietary treatment between CD, HFD, HFrD, and HFHFrD mice. Similarly, since *Clostridium* and *Bacteroides* species are involved in producing metabolites such as bile salts and SCFAs [95], with bacteria like *C. butyricum* participating in butyrate production and its role as a protector in diseases like IBD being studied [96]; or *Bacteroides* species such as *B. ovatus*, which helps hydrolyze bile salts [97], a decrease in these bacteria could lead to the accumulation of secondary bile salts. It is important to investigate in the future how exposure to obesogenic diets influences the abundance of more *Clostridium* and *Bacteroides* species, as well as their metabolite production.

## 5. Conclusions

In this study, we found that all obesogenic diets trigger and exacerbate inflammation, atrophy, and damage to the colonic epithelium in a preclinical obesity model in C57BL/6 mice. Exposure to High-Fructose diets significantly worsens intestinal inflammation, atrophy, and damage, as evidenced by a substantial increase in the IAD score in both HFrD and HFHFrD mice. The findings revealed changes in mucin production and expression, characterized by a significant increase in neutral and mixed mucins. All these events disrupt barrier function and lead to dysbiosis. However, the dysbiosis induced by each type of obesogenic diet differs only by HFD increasing the Firmicutes/Bacteroidetes and *Clostridium*/*Bacteroides* ratios in the early stages. Additionally, a notable increase in *Bacteroides,* a bacterial species known for its anti-inflammatory properties, was observed in mice fed an HFrD and HFHFrD diet early on. Even so, over prolonged periods, all obesogenic diets lead to a decrease in microbial abundance and richness. Although future studies are necessary to examine the long-term effects of obesogenic diets and to analyze changes in a greater variety of bacterial species related to bile acid and SCFA metabolism, this study provides valuable information and proposes the use of species ratios, such as *Clostridium*/*Bacteroides*, as a potential biomarker in fat-rich microenvironments. This could help us understand the dynamics of dysbiosis and its association with colon damage before the development of more severe illnesses such as inflammatory bowel disease or cancer.

## Figures and Tables

**Table 1:** Primer Sequences for qPCR of Bacterial Phyla.

Gene	Primer		
*16sRNA* *Universal RT*	F URT: 5′ R URT: 5′	TGCCAGCAGCCGCGGTAATA TGACGTCATCCCCACCTTCCTC	3′ 3′
Actinobacteria	F: 5′ R URT: 5′	CGGTGGATGCTGGATGTGG TGACGTCATCCCCACCTTCCTC	3′ 3′
Proteobacteria	F: 5′ R URT: 5′	CCTGGGAACTGCATCTGATACTGG TGACGTCATCCCCACCTTCCTC	3′ 3′
Bacteroidetes	F: 5′ R URT: 5′	TGGTGTAGCGGTGAAATGCT TGACGTCATCCCCACCTTCCTC	3′ 3′
Firmicutes	F: 5′ R URT: 5′	GCGAAGGCGGCTCTCTGG TGACGTCATCCCCACCTTCCTC	3′ 3′

Abbreviations: F = forward, R = reverse, URT = Universal Real-Time primer. For bacterial phyla, the forward primers were designed within the variable region, while the reverse primer was identical to the one used for amplifying the conserved region of the 16S ribosomal subunit, as indicated in Figure S2. All primers were designed by our team using the https://www.primer3plus.com/ platform and analyzed in https://www.bioinformatics.org/ for specific amplification. Accessed on 3 February 2024.

**Table 2:** Primer Sequences for qPCR of Bacterial Species.

Gene		Primer	
*16sRNA* *Universal RT*	F: 5′ R: 5′	TGCCAGCAGCCGCGGTAATA TGACGTCATCCCCACCTTCCTC	3′ 3′
*C. scindens*	F: 5′ R: 5′	CTGGCTCAGGAATGAACGCT CACTCCAGCCACGCAGTT	3′ 3′
*C. hiranonis*	F: 5′ R: 5′	CCTAACACATGCAAGTCGAGC CCTTGAGGACAGAGCTTTACGA	3′ 3′
*C. hylemonae*	F: 5′ R: 5′	GTCGAACGAAGCAATACTGTGTG CTCTACCATGCGGTACTGAGGT	3′ 3′
*L. acidophilus*	F: 5′ R: 5′	GCTGGCGGCGTGCCTAATACA GCGGGGCCATCCCATAGCG	3′ 3′
*B. Bifidum*	F: 5′ R: 5′	ACGGGTAGCCGGCCTGAGAG CGAGCCGCCTACGAGCCCTT	3′ 3′
*B. tethaiotaomicron*	F: 5′ R: 5′	TGCCAGCAGCCGCGGTAATA TGAGCTGCCTTCGCAATCGGA	3′ 3′
*B. fragilis*	F: 5′ R: 5′	GGCGCTAGCCTGAACCAGCC ACCAGTCCACCTACGCTCCCT	3′ 3′
*E. coli*	F: 5′ R: 5′	TTGCTGACGAGTGGCGGACG CCCCACTGCTGCCTCCCGTA	3′ 3′

Abbreviations: F = forward, R = reverse. For bacterial species, the forward and reverse primers were designed within the variable region of the 16S ribosomal subunit. All primers were designed by our team using the https://www.primer3plus.com/ platform and analyzed in https://www.bioinformatics.org/ for specific amplification.

## Data Availability

The original contributions presented in the study are included in the article/supplementary material, further inquiries can be directed to the corresponding author.

## Supplementary Materials

The following supporting information can be downloaded at: https://www.mdpi.com/article/10.3390/nu18152471/s1, Supplementary Figure S1. (A) Food intake per week for each group of mice exposed to different obesogenic diets and the control diet. (B) Kilocalories consumed per week by the four groups of mice fed obesogenic diets and the control group. (C) Average food intake per group of five mice exposed to obesogenic and control diets. (D) Average kilocalories consumed per group of five mice exposed to obesogenic and control diets. Asterisks (*) indicate statistically significant differences between groups, with * *p* < 0.05, ** *p* < 0.01, and *** *p* < 0.001. Supplementary Figure S2. For each of the following phyla: Firmicutes, Bacteroidetes, Actinobacteria, and Proteobacteria; the forward primers were designed in the variable region, while the reverse primer was the same one used for amplifying the conserved region of the 16S ribosomal subunit. Supplementary Table S1. Caloric content and composition of diets. Supplementary Table S2. Parameters for Determining the IAD Score.
