# Supplementary material for: Obesogenic Diets Composition Differentially Alters the Clostridium/Bacteroides Ratio and Drives Colonic Inflammation
Source: Nutrients. 2026 Jul 30;18(15):2471. doi: 10.3390/nu18152471 (PMC13468517; doi:10.3390/nu18152471)
Supplement: Supplementary file 1 [file nutrients-18-02471-s001.zip › nutrients-4344722-supplementary.pdf]

Supplementary Table S1. Caloric content and composition of diets

	CD		HFD		HFrD		HFHFrD	
	g%	kcal%	g%	kcal%	g%	kcal%	g%	kcal%
**Protein**	24.1	28.9	26.2	20	24.2	29.3	26.3	20.4
**Carbohydrate**	53.4	57.4	26.3	20	138.2	396.6	111.1	359.2
**Fat**	11.5	13.6	34.9	60	11.8	16.3	35.2	62.7
**Kcal/g**	3.35		5.24		6.77		8.66	

The table presents a comparison of the caloric content provided by proteins, carbohydrates, and fats for each diet. Control Diet (CD), High-Fat Diet (HFD), High-Fructose Diet (HFrD), and High-Fat and High-Fructose Diet (HFHFrD).

Supplementary Table S2. Parameters for Determining the IAD Score

Inflammatory cell infiltrate			Intestinal architecture		
**Severity**	**Extension**	**Score 1**	**Epithelial changes**	**Mucosal architecture**	**Score 2**
Null	Null extension	0	Intact epithelium	No changes	0
Mild	Mucosal	1	Minimal Hyperplasia	No changes	1
Moderate	Mucosal, sometimes submucosal	2	Mild hyperplasia, minimal goblet cell loss, with or without erosions.	Mild loss of architecture	2
Severe	Mucosal, submucosal, and occasionally transmural	3	Moderate hyperplasia, some crypt abscesses, mild goblet cell loss, with or without erosions.	Focal ulcerations	3
Severe	Transmural	4	Severe hyperplasia, moderate goblet cell loss.	Extensive ulceration	4

The criteria for assessing the degree of intestinal inflammation, atrophy, and damage (IAD score) were adapted from Erben et al., 2014, with slight modifications [33]

Supplementary Figure S1

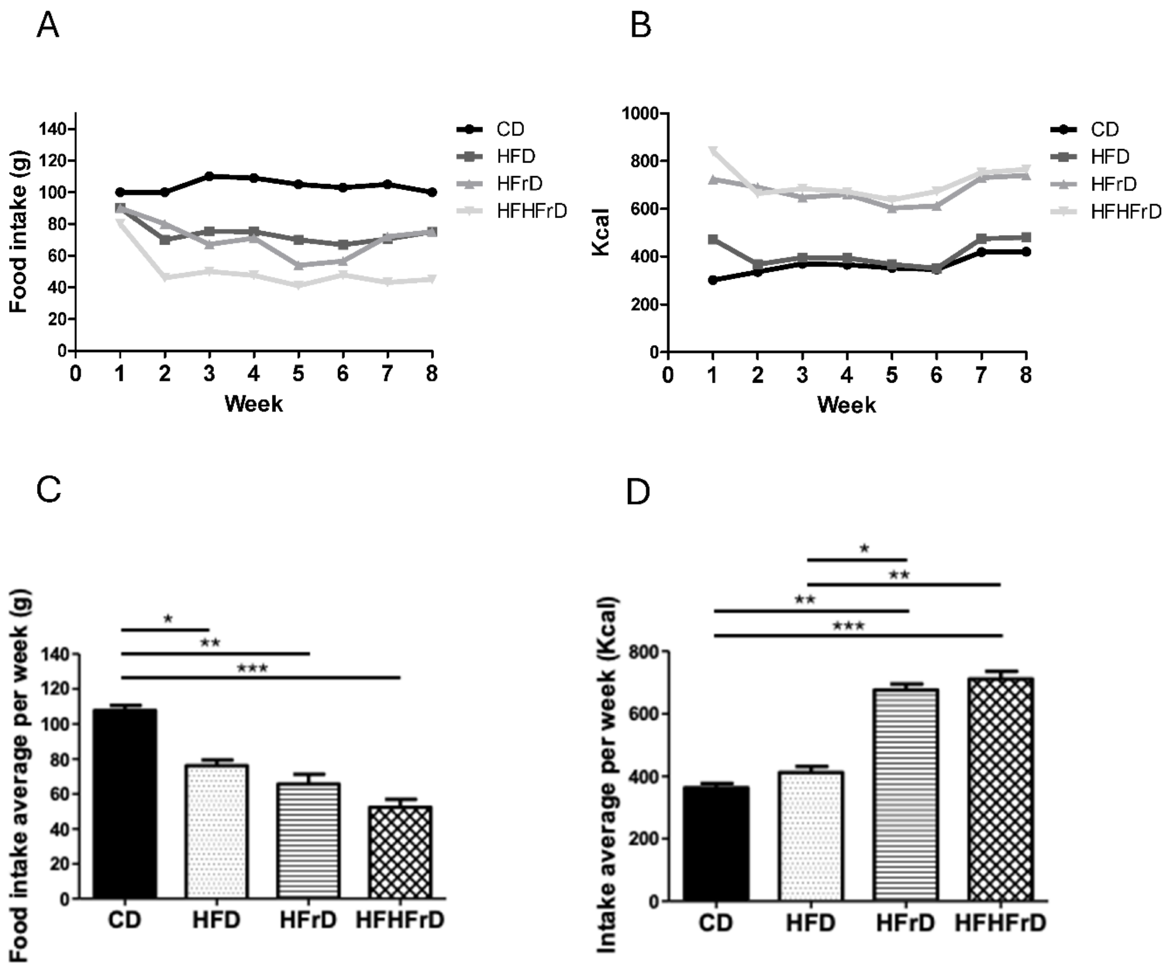

Supplementary Figure S1. A) Food intake per week for each group of mice exposed to different obesogenic diets and the control diet. B) Kilocalories consumed per week by the four groups of mice fed obesogenic diets and the control group. C) Average food intake per group of five mice exposed to obesogenic and control diets. D) Average kilocalories consumed per group of five mice exposed to obesogenic and control diets. Asterisks (*) indicate statistically significant differences between groups, with *$p < 0.05$, **$p < 0.01$, and ***$p < 0.001$.

Supplementary Figure S2

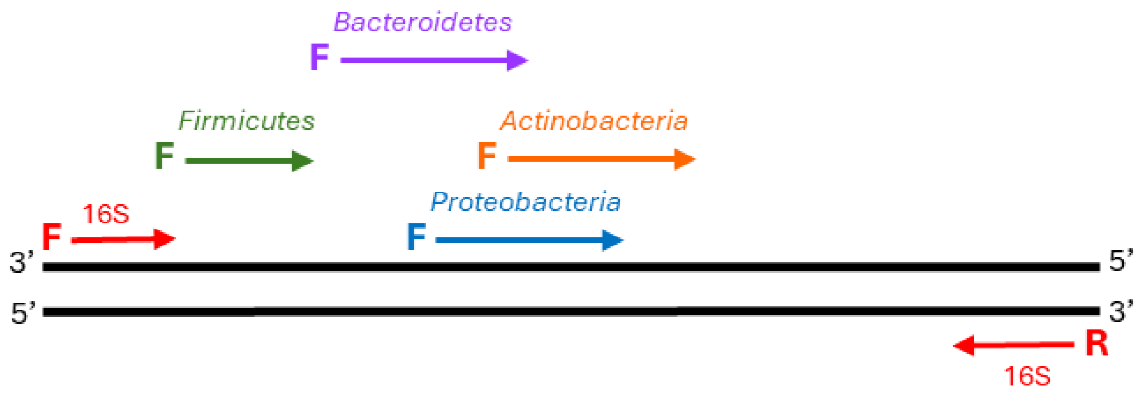

Supplementary Figure S2. For each of the following phyla: Firmicutes, Bacteroidetes, Actinobacteria, and Proteobacteria; the forward primers were designed in the variable region, while the reverse primer was the same one used for amplifying the conserved region of the 16S ribosomal subunit.
